# Supplementary material for: Exploring the Bioactive Potentials of C60-AgNPs Nano-Composites against Malignancies and Microbial Infections
Source: Int J Mol Sci. 2022 Jan 10;23(2):714. doi: 10.3390/ijms23020714 (PMC8776077; doi:10.3390/ijms23020714)
Supplement: Supplementary file 1 [file ijms-23-00714-s001.zip › ijms-1445278-supplementary.pdf]

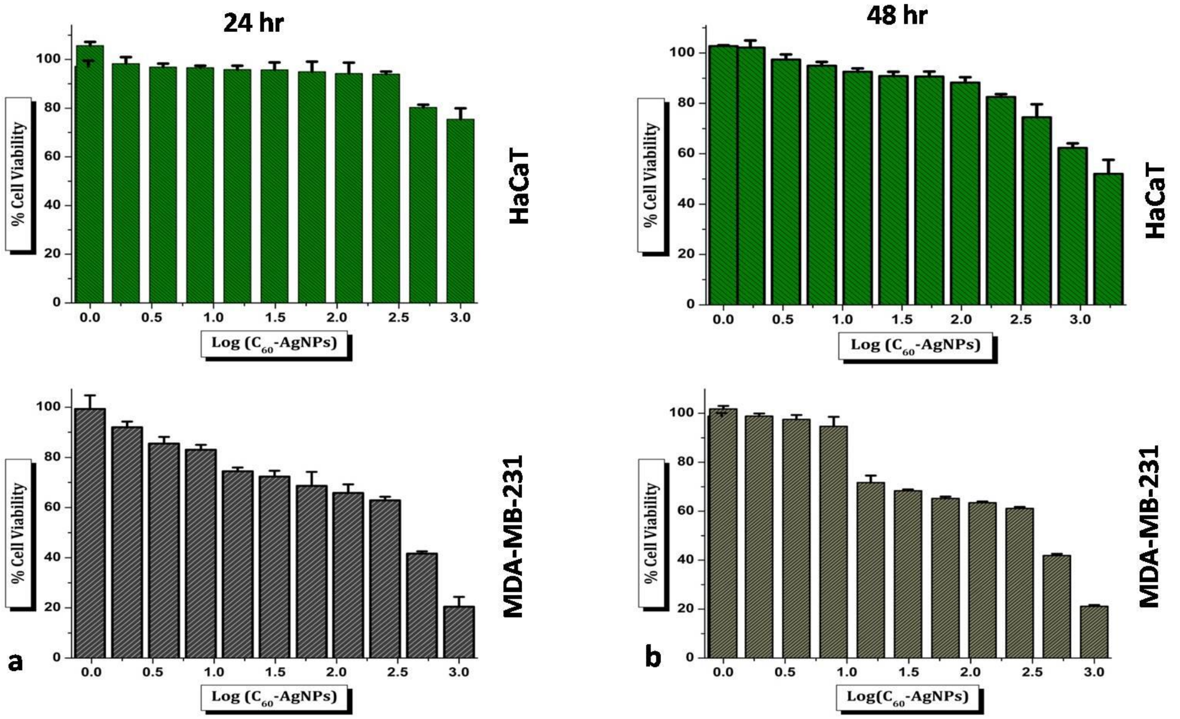

Figure S1. Cell viability assay of (**a**) C_{60}-AgNPs nanocomposite treated HaCaT&MDA-MB-231 cell lines [24-hour post incubation] and (**b**) C_{60}-AgNPs nanocomposite treated HaCaT&MDB-MB-231 [48-hour post incubation].
